# Quantitative real-time PCR assays for species-specific detection and quantification of Baltic Sea spring bloom dinoflagellates

**DOI:** 10.3389/fmicb.2024.1421101

**Published:** 2024-09-24

**Authors:** Annica Marie Brink, Anke Kremp, Elena Gorokhova

**Affiliations:** ^1^Department of Ecology, Environment and Plant Sciences, Stockholm University, Stockholm, Sweden; ^2^Biological Oceanography, Institute for Baltic Sea Research Warnemünde, Rostock, Germany; ^3^Department of Environmental Science and Analytical Chemistry, Stockholm University, Stockholm, Sweden

**Keywords:** Baltic Sea, spring bloom, dinoflagellates, *Apocalathium malmogiense*, *Biecheleria baltica*, *Gymnodinium corollarium*, qPCR, real-time PCR

## Abstract

In the Baltic Sea, the dinoflagellates *Apocalathium malmogiense*, *Biecheleria baltica*, and *Gymnodinium corollarium* are important contributors to the spring bloom. However, their relative contribution to the bloom community cannot be unambiguously determined by conventional light microscopy due to a lack of resolution of distinctive morphological features of the three species. Here, we describe a molecular approach based on a quantitative real-time polymerase chain reaction (qPCR) primer and probe system, targeting the ITS1 and ITS2 regions of the rRNA gene for all three species and enabling their quantification. The specificity of the method was demonstrated using monocultures of *A. malmogiense*, *B. baltica*, *G. corollarium* as well as three other dinoflagellate species co-occurring in the Baltic Sea during spring and validated using field-collected phytoplankton samples.

## 1 Introduction

Dinoflagellates are arguably the largest group of marine eukaryotic phytoplankton aside from diatoms, and one of the most important primary producers in the marine ecosystem. Cold-water dinoflagellates are an important component of the algal spring bloom in the Baltic Sea (Lignell et al., 1993; Tallberg and Heiskanen, 1998). During the last four decades, their proportion has increased significantly in some regions, and dinoflagellates now frequently dominate the spring bloom in the central and northern basins of the Baltic Sea (Klais et al., 2011; Spilling et al., 2018). Recent morphological and molecular analyses revealed that in addition to the chain-forming arctic *Peridiniella catenata*, at least three other species are associated with the spring dinoflagellate blooms in the Baltic (Larsen et al., 1995; Kremp et al., 2005; Sundström et al., 2009). The re-described *Biecheleria baltica* Moestrup, Lindberg et Daugbjerg, formerly known as *Woloszynskia halophila* (*sensu* Kremp et al., 2005) is common in the Gulf of Finland, co-occurring with *Apocalathium malmogiense* (G. Sjöstedt) Craveiro, Daugbjerg, Moestrup & Calado, formerly known as *Scrippsiella hangoei* (*sensu* Larsen et al., 1995) (Craveiro et al., 2017). The third species, *Gymnodinium corollarium* Sundström, Kremp et Daugbjerg (Sundström et al., 2009), occurs throughout the Baltic Sea and is particularly abundant in the open Baltic proper (Sundström et al., 2009). Thus, all three species co-occur in the northern Baltic Sea and likely in other regions, both within and outside of the Baltic Sea.

Due to their similar size and gross appearance, these species cannot be unambiguously distinguished from one another by light microscopy with or without staining, particularly in samples preserved with Lugol iodine solution (Kremp et al., 2005; Sundström et al., 2009). Unlike *B. baltica* and *G. corollarium*, *A. malmogiense* is armored; therefore, staining with CalcoFluor White could be applied to visualize the thecal plate patterns (Fritz and Triemer, 1985). However, the thecal plates in this species are delicate and stain poorly, thus hampering the identification (Larsen et al., 1995). Cell surface features specific for unarmored *B. baltica* or *G. corollarium* can only be observed using scanning electron microscopy (SEM), a method not applicable for quantitative enumeration of algae in field plankton samples.

The difficulties with species identification are hampering our ability to study population dynamics of the individual dinoflagellate species and, thus, understand the seasonal succession of these ecologically important Baltic phytoplankton species. To understand whether phenology and bloom magnitude are driven by life-history traits and environmental factors involved in the life cycle regulation in *A. malmogiense*, *B. baltica*, and *G. corollarium* (Kremp et al., 2005), we need to distinguish these species during the analysis. For instance, research on the formation and germination of cysts, initially attributed to *Scrippsiella hangoei*/now named *A. malmogiense*, but later identified as those of *B. baltica*, has shown that the seasonal occurrence of this species is governed by life cycle changes influenced by physiological and environmental factors (Kremp and Anderson, 2000). Moreover, species-specific encystment strategies might influence biogeochemical processes in the sediment once the bloom has settled out from the water column (Spilling and Lindström, 2008). Furthermore, variations in the relative contribution of these species to the spring bloom may affect their grazers. For example, interspecific differences in the allocation of fatty acids (Leblond et al., 2006) may lead to higher egg production in copepods fed *G. corollarium* (Vehmaa et al., 2012) with consequences for energy transfer efficiency in the food web. Similarly, stoichiometry and sterol production variability among the dinoflagellate species (Chen et al., 2019) may influence the dinoflagellate-grazer relationships and zooplankton growth and dynamics (Thomas et al., 2022). To study all these ecological processes, we need reliable methods for population analysis of these cold-water dinoflagellates co-occurring in the Baltic Sea.

In the last decades, various molecular assays [e.g., real-time PCR, fluorescent hybridization assay, high resolution melting (HRM), sandwich hybridization assay, etc.] were shown to be instrumental for the identification and enumeration of dinoflagellates, improving the detection capacity and reducing sample processing time. Earlier, a molecular identification method based on fluorescence *in situ* hybridization (FISH) assay has been developed for *B. baltica* (Sundström et al., 2010). The method was successfully applied to study the seasonal succession of this dinoflagellate at the SW coast of Finland. However, the labeling efficiency was affected by physiological changes in the algae during the bloom due to nutrient limitation, growth phase, and life cycle transitions; therefore, the quantification was inherently uncertain (Sundström et al., 2010).

**Figure 1:**
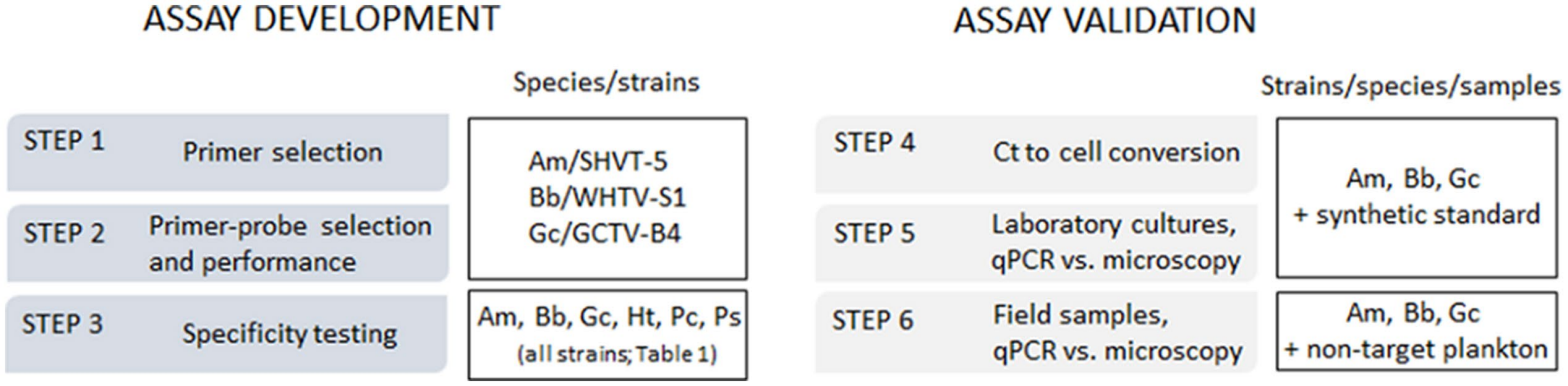
The outline of the qPCR assay development and validation steps and species/strains used for specific tasks. Am, *Apocalathium malmogiense*; Bb, *Biecheleria baltica*; Gc, *Gymnodinium corollarium*.

The aim of this study was to develop TaqMan qPCR assays for the identification and enumeration of *A. malmogiense*, *B. baltica*, and *G. corollarium* in Baltic Sea plankton. The emphasis was on ensuring these methods were applicable to regular monitoring surveys, including their use with plankton samples collected and preserved according to the standard monitoring guidelines in the region (HELCOM, 2017). To confirm the assay specificity, each primer-probe set was tested not only with monocultures of *A. malmogiense*, *B. baltica*, and *G. corollarium*, but also with three other dinoflagellate species co-occurring in the Baltic Sea with the target species during spring. The assay development proceeded in a step-wise manner and was validated using field-collected samples (Figure 1).

## 2 Materials and methods

### 2.1 Test strains and environmental samples

#### 2.1.1 Cultures

For the assay development, we used cultures of *A. malmogiense* (four strains), *B. baltica* (four strains), *G. corollarium* (three strains), and other dinoflagellates that had been isolated from samples collected at the SW coast of Finland and have been maintained at the Tvärminne Algal Culture collection, now included in the Algal Culture Collection of SYKE Marine Research Centre (Table 1 and Figure 1). Except for *Protodinium simplex*, which was ordered from SCCAP (Denmark) and preserved with Lugol iodine solution upon arrival, all cultures used in this study (Table 1) were grown in temperature-regulated incubators at 50 μmol photons m^−2^ s^−1^ and a 14:10 h light:dark cycle. As a growth medium, F/2-Si enrichment (Guillard and Ryther, 1962) at 6.5 salinity was used; throughout this paper, the salinity values are given in practical salinity units (PSU). All cultures were grown at 4°C, except for *Heterocapsa triquetra*, which was maintained at 17°C. Subsamples of the cultures in the exponential growth phase were taken, preserved with Lugol iodine solution and stored chilled in the dark until they were used for microscopy analysis and DNA extraction.

**Table 1:** Cultures of dinoflagellate species and strains used in this study.

Species	Strain	Origin
*Apocalathium malmogiense*	SHTV^b^	Tvärminne (SW coast of Finland)
*Apocalathium malmogiense*	SHTV-0910^b^	Station Storfjärden, Tvärminne (SW coast of Finland)
*Apocalathium malmogiense*	SHTV-0908^b^	Station Storfjärden, Tvärminne (SW coast of Finland)
*Apocalathium malmogiense*	SHTV-5^b^	Tvärminne (SW coast of Finland)
*Biecheleria baltica*	WHTV^a^	Tvärminne (SW coast of Finland)
*Biecheleria baltica*	WHTV-15^a^	Tvärminne (SW coast of Finland)
*Biecheleria baltica*	WHTV-S8^a^	Station BY31, Northern Baltic Proper
*Biecheleria baltica*	WHTV-S1^a^	Northern Baltic Proper, Sweden
*Gymnodinium corollarium*	GCTV-B4	Station BY29, Northern Baltic Proper
*Gymnodinium corollarium*	GCTV-03	Station BY15 (Gotland deep), Baltic Proper
*Gymnodinium corollarium*	GCTV-A3	Station BY29, Northern Baltic Proper
*Heterocapsa triquetra*	HTF1002	Föglö, Åland Archipelago, Finland
*Peridiniella catenata*	PCTV0907	Station Storfjärden, Tvärminne (SW coast of Finland)
*Protodinium simplex*	K-0661	English Channel, UK/SCCAP (Denmark)

a The strain abbreviation is based on the former name of the species, *Woloszynskia halophila* (Kremp et al., 2005).

b The strain abbreviation is based on the former name of the species, *Scrippsiella hangoei* (Larsen et al., 1995).

#### 2.1.2 Field samples

In addition to the monocultures, field samples were collected in a coastal area (Himmerfjärden Bay, 58°.59′N, 17°.43′E; bottom depth 32 m) and used for the assay validation (Figure 1). Two time periods were selected for the sample collection to ensure (1) the presence of abundant and diverse dinoflagellate communities (March 15–June 8, 2010), when all target species are present ubiquitously in the water column; and (2) the absence of any of the target species in the plankton (September 28, 2010); by this time all of them should have undergone complete encystment and disappear from the water column (Kremp et al., 2009). All samples were collected in the photic zone (0–14 m) during the daytime using a plastic hose (inner diameter of 25 mm), and the entire sample volumes were transferred to a bucket. After thorough mixing, two subsamples of the collected plankton were drawn from each sample and designated for (1) microscopy analysis of the *Apocalathium*/*Biecheleria*/*Gymnodinium* complex; these samples (200 mL) were immediately preserved with acetic Lugol iodine solution (0.8 mL), and (2) qPCR assay; these samples (1 L) were also preserved with Lugol iodine (4 mL). All samples were stored chilled in darkness until analyses.

### 2.2 Microscopy analysis

Subsamples (25 mL) of the phytoplankton samples designated for microscopy-based identification and enumeration of dinoflagellates were settled for 20–40 h in Utermöhl chambers following the guidelines for phytoplankton analysis in the Baltic Sea (HELCOM, 2017). Dinoflagellates were counted on the half-bottom or entire bottom of the sedimentation chamber under an inverted light microscope (Leitz DM-II) at 200× or 400× magnification. All counts were conducted in triplicate. Cell concentrations were calculated from obtained counts (cell mL^−1^) and used for the preparation of dilution series in qPCR assays, in the calculations of rRNA gene copy number per cell, and as an independent estimate of cell abundance.

### 2.3 Design and verification of specific primers and probes

#### 2.3.1 The qPCR approach

For each species, we designed primers and a hydrolysis probe (minor groove binder, MGB), which carries a fluorophore at the 5′-end and a quencher at the 3′-end; see Supplementary Text S1. The hybridized probe is cleaved by the 5′ exonuclease activity of the Taq polymerase resulting in fluorescence increase. The species-specific primers and those probe sequences characterized by the highest number of mismatches to non-target sequences were tested *in silico* for their specificity by a BLAST sequence similarity search^1^ against the GenBank nucleotide collection. Due to the additional specificity provided by the presence of the TaqMan probe, this approach was selected because it is less subject to false positives than the intercalating dye method (Kutyavin et al., 2000), which is crucial for mixed plankton samples.

#### 2.3.2 Sequence information

The target for designing primers and probes was the rDNA ITS, regions ITS1 and ITS2. Sequencing of *B. baltica* (strain WHTV-C1) and *G. corollarium* (strain GCTV-B4) were performed in collaboration with the Department of Biomolecular Sciences, University of Urbino, Italy. The 5.8S rRNA gene and flanking ITS regions were amplified and sequenced using the BigDye Terminator Cycle Sequencing Kit (Applied Biosystems, Cheshire, United Kingdom) and an ABI PRISM 310 Genetic Analyzer instrument. The sequences have been deposited into the GenBank database (accession numbers MN525428 and MN525429 for *G. corollarium* GCTV-B4, and *B. baltica* WHTV-C1, respectively). For *B. baltica*, WHTV-C1 sequence and GenBank sequence DQ167868 were used to generate a consensus sequence. For *A. malmogiense*, a consensus sequence was created based on the GenBank sequences for the following strains: SHTV-2 (AY970655), SHTV-5 (AY970656), SHTV-6 (AY970657), SHTV-1 (AY499515), and 702 (EF205037).

#### 2.3.3 qPCR primer and probe design

The primer-probe systems (Supplementary Table S1) were first tested *in silico* for specificity. For each target species, alignments were made using the software BioEdit Sequence Alignment Editor version 7.0.9.0 (Hall, 1999). Non-target species were included based on taxonomic relatedness to the target species, strong sequence match, and occurrence of the species in the Baltic Sea spring phytoplankton community. The alignments were searched manually to determine unique sequences within the ITS regions relevant for primer and probe design, which was then performed using Primer3 (Whitehead Institute and Howard Hughes Medical Institute, Maryland). A database similarity search was made using BLAST to ensure *in silico* specificity of primers and probes. Primers were synthesized by Sigma-Aldrich Sweden AB (Stockholm, Sweden) and probes by Applied Biosystems (Cheshire, United Kingdom). The probes were dual-labeled with the fluorophore 6FAM and the quencher MGBNFQ at the 5′ and 3′ ends, respectively.

### 2.4 DNA extraction and quantification

#### 2.4.1 DNA extraction

The DNA was obtained by Chelex extraction (Giraffa et al., 2000) using Lugol-preserved samples of dinoflagellates and wild plankton; this method was selected based on pilot tests evaluating several extraction protocols (Supplementary Text S2). For all extractions, the appropriate culture/sample volume was filtered onto a 5.0 μm Millipore TMTP Isopore polycarbonate membrane filter (Ø 25 mm) (Millipore, MA, United States) using a 15 mL Millipore filtration tower (Ø 16 mm) fitted to a vacuum filtration manifold. The filter was placed in a petri dish and cut diagonally with a scalpel into eight pieces which were all placed in a 2 mL tube. Nuclease-free water (200 μL; Qiagen, Hilden, Germany) was added, and samples were centrifuged at 10,000 rpm for 5 min, and then frozen at −80°C; see Supplementary Text S2 for the details and justification of this procedure. After thawing, each sample received approximately 100 mg of acid-washed glass beads (≤106 μm; Sigma-Aldrich, MO, United States) and was homogenized for 2 × 20 s in a FastPrep^®^-24 Instrument (MP Biomedicals, CA, United States). Then, 200 μL Chelex^®^ 100, 10% w/v (Bio-Rad, CA, United States) were added followed by heating at 50°C for 30 min and then at 105°C for 8 min. The sample was centrifuged at 10,000 rpm for 5 min, and the supernatant (100 μL) containing the DNA was transferred into a 1.5 mL tube and stored at 8°C overnight before processing.

#### 2.4.2 DNA yield and purity assessment

The quantity of the extracted DNA was evaluated by reading the whole absorption spectrum (220–750 nm) with a Nanophotometer^™^ (Implen), and the DNA quality was assessed using absorbance ratio at both 260/280 and 230/260 nm. Both ratios were acceptable in most of the samples (1.8–2.0 and 2.0–2.2, respectively). In addition to the concentrations measured spectrophotometrically, the DNA concentration in each sample was also quantified fluorometrically by staining with Hoechst dye 33258 (Sigma-Aldrich) to avoid an overestimation of the DNA concentration measured by Nanophotometer (Bhat et al., 2010). The fluorometry-based DNA concentrations were applied to estimate template concentration in the TaqMan real-time PCR reaction.

### 2.5 DNA standards and qPCR standard curves

We used and compared synthetic oligonucleotide-based and cell-based standard curves to provide a comprehensive approach to qPCR quantification. The synthetic oligonucleotide standards ensured precise control over initial DNA concentrations and sequence specificity, while the cell-based curves accurately reflected real-world conditions, including extraction efficiencies and sample complexity. This dual approach allowed us to validate and enhance the reliability of our quantitative results across different aspects of the qPCR workflow.

#### 2.5.1 Synthetic standards for qPCR

**Figure 2:**
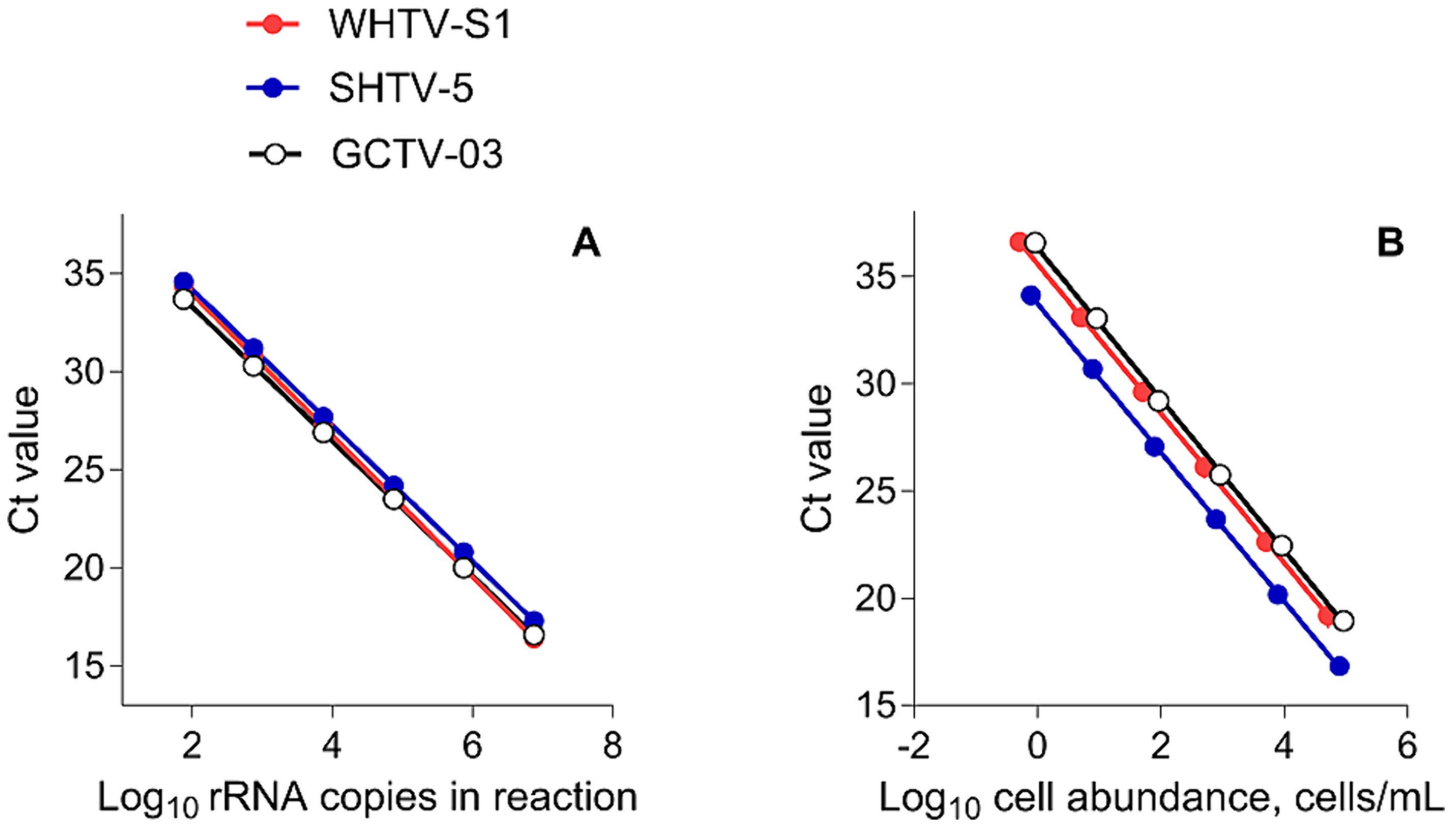
Standard curves of **(A)** Log_10_ starting rRNA gene copy number in the reaction vs. the threshold cycle number (Ct) for qPCR detection of DNA from synthetic controls in the gene-based assays (*n* = 6) and **(B)** cell-based assays for the target species/strains. All plots show mean values; the standard deviation bars are not visible (*n* = 5).

Gene-based standard curves were constructed for all three species to determine the assay’s efficiency, using a synthetic gene fragment approach (Vermeulen et al., 2009). A synthetic DNA oligonucleotide (Invitrogen Ltd.) comprising the target sequences (Table 2) and cloned into a plasmid was used as a standard (Figure 2). For each target species, the synthetic gene was assembled and cloned into pMA-T plasmid using *Sfil* and *Sfil* cloning sites. The plasmid DNA was purified from transformed bacteria (*E. coli*, K-12), and concentration was determined by UV spectroscopy. The final construct was verified by sequencing, and the congruence within the restriction site was 100%. When preparing the DNA for the standard dilutions, 5 μg of the lyophilized plasmid DNA were dissolved in 50 μL distilled water. For the standard curves based on the synthetic gene fragments, a 10-fold serial dilution ranging 75 to 75 × 10^5^ copies per reaction for all species of the plasmid construct containing the target sequence, were generated and used as a template. Each reaction was performed in duplicate.

**Table 2:** Synthetic oligonucleotides used as standards for *Apocalathium malmogiense* (ITS2), *Biecheleria baltica* (ITS1), and *Gymnodinium corollarium* (ITS2) in the qPCR assays.

Target species	Primer (forward/reverse) and probe set	Oligo sequence (5′ → 3′)	Size of the standard (bp)
*Apocalathium malmogiense*	S459F/S484 + S542R	CAATCGTCCTTGACGCATTCAGAGCATGGG**GATTTCATCTGGTCGCA** **CAA**CGAATCATACATCTCTGATGTTGCTTGTTGGTGCATGTCGAACA	94
*Biecheleria baltica*	B51F/B169+ B211R	CAATCATGTGAGTGACTGGGTGGAGATGGTTGCGCTCCGCGCGCAC TTCCTCCATGTGGGAGCTCGCGGGTGGCAGGGCAGCCTGGCAACGC GTGTCGTTCCTTGTGTGCGGCGCGGTGGCCC**AGGTTGTTCCTGTTGC** **CATT**GTGTTTGCTCTGGCTCAACTGTCGAACA	171
*Gymnodinium corollarium*	G458F/G535 + G602R	CAATCGCGCAGTGTCTACCTTCGTGTGGGCCATGGTGCTCCTGAGGC ATTTGATTCACAGGGTCCTGCTGCGACCGCCAGCT**TACTGAGCATCTC GGTGTGC**GGCTAGCTGCGCGTGTTGTAAATAGTGCCCTGTCTTCTCAC GGCTCTGGAACA	155

The oligo DNA includes sequence between the forward and the reverse primers (underlined) and the corresponding probe in-between (bold); CAATC and GAACA (grey letters) were added at the 5′ and 3′-end, respectively. The oligonucleotides were synthesized by Invitrogen (Life Technologies) and cloned into pMA-T plasmid (Figure 2).

#### 2.5.2 Cell-based standard curves and determination of gene copy number per cell

One strain of each species, *A. malmogiense* (SHTV-5), *B. baltica* (WHTV-S1), and *G. corollarium* (GCTV-B4), were used to extract genomic DNA and construct cell-based standard curves. These extractions were serially diluted and used to generate target DNA representing a known cell number per reaction versus Ct data. The cell-based standard curves were constructed using 10-fold dilutions of the DNA extract using known cell concentrations ranging 1 to 8 × 10^3^ cells mL^−1^ for *A. malmogiense*, 1 to 7 × 10^3^ cells mL^−1^ for *B. baltica*, and 1 to 9 × 10^3^ cells mL^−1^ for *G. corollarium*. Each reaction was performed in triplicate.

#### 2.5.3 Determination of gene copy number per cell

To determine the mean number of the rRNA gene copies per cell, the dilution series with a known cell count and gene-based Ct values were used as input for calculation. The slope of the linear regression between the rRNA gene copies and cell numbers in the reaction were used to determine the copy number per cell. This analysis was conducted for three strains of each target species (Table 1).

### 2.6 Assay performance

#### 2.6.1 Primer performance

Candidate primers for *A. malmogiense*, *B. baltica*, and *G. corollarium* were tested using conventional PCR and DNA extracts from the monocultures of SHTV-5, WHTV-S1, and GCTV-B4, respectively. Each PCR reaction contained 2 μL of extracted DNA, 5 μL of Standard Taq Reaction Buffer (Final concentration 2.5×, 3.75 mM MgCl2, New England BioLabs, MA, United States), 1 μL of dNTPs (Final concentration 0.5 mM, New England BioLabs), 0.5 μL of Taq polymerase (corresponding to 2.5 units, New England BioLabs), 2 μL of each primer (final concentrations 1 μM), and 7.5 μL of nuclease-free water (Qiagen, Hilden, Germany) resulting in a total reaction volume of 20 μL. Nuclease-free water was used instead of DNA for the non-template controls (NTC). Samples were amplified using MJ Research MiniCycler for 2 min at 94°C followed by 30 cycles of 15 s at 94°C, 15 s at 60°C and 45 s at 72°C with a final extension step for 7 min at 72°C. PCR products were mixed with 6× loading dye solution and ran on a 1.5% agarose gel. GeneRuler 100 bp DNA Ladder (Thermo Scientific, MA, United States) was used as a marker. The gel was stained with ethidium bromide (0.2 μg/L) for 20 min, rinsed in distilled water for 7 min and visualized under UV light. All gels were examined to ensure that the PCR products had the expected size (bp) and no amplification occurred in NTC. Two main criteria for primer selection for qPCR were used: (1) efficient amplification as indicated by clear and sharp bands in the gels, and (2) the product size, with shorter amplicon size being favored as more suitable for qPCR (Penna and Galluzzi, 2013).

#### 2.6.2 Primer-probe performance

To ensure proper binding to the target species, functional primers with the corresponding probes were tested by qPCR using a StepOne Real-Time PCR instrument (Applied Biosystems). The following reagents were added for a 20 μL reaction mixture: 10 μL of TaqMan Gene Expression Master Mix (Applied Biosystems), 1.5 μL of each primer (0.75 μM), 0.5 μL of the probe (0.25 μM), 2 μL of the DNA extracted from a target species, and 4.5 μL of nuclease-free water. For the NTC, nuclease-free water was used instead of DNA. The thermal cycling conditions consisted of 2 min at 50°C and 10 min at 95°C followed by 40 cycles of 15 s at 95°C and 1 min at 60°C. Fluorescence data were collected at the end of each cycle, and the cycle threshold was determined automatically by the instrument software. Extracted DNA from the cultures SHVT-5, WHTV-S1 and GCTV-B4 was used in six-step tenfold serial dilutions (1×–10^5^×) and primer-probe sets were evaluated based on the qPCR efficiency and correlation coefficients (*R*^2^). All samples, standards, and NTC were analyzed in triplicates.

#### 2.6.3 Reproducibility of the standard curve assays

The method reproducibility (inter-assay variation) was assayed by calculating the CV_Ct_ (coefficient of variation for cycle threshold) of gene-and cell-based standards in independent experiments run on different days using different sets of dilutions; five assays were used for gene-based standard curves, and four assays were used for cell-based standard curves. The replicate standard curves were produced with the final set of primers and probes using a serial dilution series over the six-step tenfold serial dilutions (1×–10^5^×). The inter-run variation was calculated for Ct values as described by Rutledge and Côté (2003). For each primer-probe set the pooled slope and intercept were calculated and used for downstream applications of the gene-based standard curve.

#### 2.6.4 Specificity testing

Each assay was tested with several strains of the respective target species (Table 1). The specificity of each assay was confirmed by using DNA from the non-target species as a template. More specifically, cross-reactivity between primers and probe for *A. malmogiense* were tested with DNA extracted from the other two species, and the same was done for the assays developed for *B. baltica* and *G. corollarium*. The specificity of each primer-probe set was further tested by screening against non-target species *Heterocapsa triquetra*, *Peridiniella catenata* and *Protodinium simplex* (Table 1) that are phylogenetically close to the target species (Saldarriaga et al., 2001; Zhang et al., 2007). All primer-probe sets were also tested with DNA from a field sample collected in spring 2010—a time when all target species were expected to be present in the water column. The qPCR products were sequenced (KIGene, Karolinska University Hospital, Stockholm, Sweden) to confirm the specificity of the assays. Furthermore, a field sample collected in the fall, not containing the target species, was also used in the qPCR assay. As positive controls, the target species samples were included in all assays.

### 2.7 Statistical analysis

Data analysis was conducted with the GraphPad software, version 7 (GraphPad Software Inc.). The linear relationships were evaluated using *R*^2^ coefficient and *F*-test was used to determine the overall significance of the regression. The regressions were compared with no assumption of the homogeneity of variances (Smithson, 2012).

## 3 Results

### 3.1 Optimization of primer-probe performance with conventional PCR and qPCR assays

#### 3.1.1 Apocalathium malmogiense

Out of seven primer pairs (Supplementary Table S1), one pair (S416F/S494R) produced an unspecific product in the NTC. All other pairs amplified the desired DNA region with no amplification in the NTC. However, the pair S366F/S542R was eliminated from further testing because of the relatively large amplicon, which was considered suboptimal for qPCR. The remaining five pairs were combined with probes and tested in the qPCR assays. Among those, the best reaction efficiency (94.8) and *R*^2^ (~1.00) were observed in the qPCR assay with primers S459F/S542R in combination with probe S484 (Table 2).

#### 3.1.2 Biecheleria baltica

Out of six primer pairs (Supplementary Table S1), two pairs (B86F/B211R and B504F/B601R) produced unspecific products in the NTC and were eliminated from further testing. The remaining four pairs amplified the target region with no product in the NTC. Two of these, B51F/B211R and B134F/B211R, were chosen for downstream qPCR testing in combination with compatible probes (B169 in both cases). In the qPCR tests, the superior efficiency and *R*^2^ values, 93.3 and ~1.00, respectively, were obtained for the primers B51F/B211R (Table 2).

#### 3.1.3 Gymnodinium corollarium

All four primer pairs (Supplementary Table S1) amplified the desired target region with no amplification in the NTC. In the qPCR assay, the primer set G458F/G602R with G535 probe produced standard curves with the highest efficiency (96.9) and *R*^2^ = 0.99 (mean values for both parameters, Table 2). In no case was a positive amplification observed in the NTC of qPCR.

### 3.2 Overall performance and reproducibility of the standard curve assays

For both gene-and cell-based assays, the standard curves were highly reproducible with high linear range (Figure 2), efficiency >90% (with very few exceptions; Tables 3, 4), and low inter-assay variation assayed by CV_Ct_ (Tables 5, 6).

**Table 3:** Gene-based standard curves for *Apocalathium malmogiense*, *Biecheleria baltica*, and *Gymnodinium corollarium* generated on separate occasions (*n* = 5).

	Assay 1	Assay 2	Assay 3	Assay 4	Assay 5
*Apocalathium malmogiense*, SHTV-5					
Slope	−3.55 ± 0.05	−3.42 ± 0.08	−3.46 ± 0.02	−3.46 ± 0.03	−3.43 ± 0.02
Intercept	41.57 ± 0.22	40.96 ± 0.37	41.25 ± 0.11	40.97 ± 0.18	40.84 ± 0.10
Efficiency	91.30	96.11	94.32	94.26	95.39
*R* ^2^	0.998	0.995	1.000	1.000	1.000
Pooled slope = −3.47 (*p* = 0.17). Pooled intercept = 41.14 (*p* = 0.07)					
*Biecheleria baltica*, WHTV-S1					
Slope	−3.49 ± 0.02	−3.51 ± 0.06	−3.67 ± 0.07	−3.54 ± 0.07	−3.55 ± 0.12
Intercept	40.28 ± 0.14	40.90 ± 0.30	41.48 ± 0.35	41.04 ± 0.36	40.86 ± 0.60
Efficiency	93.31	92.58	87.29	91.55	91.23
*R* ^2^	0.999	0.997	0.996	0.997	0.987
Pooled slope = −3.60 (*p* = 0.37). Pooled intercept = 41.13 (*p* = 0.08)					
*Gymnodinium corollarium*, GCTV-B4					
Slope	−3.41 ± 0.02	−3.39 ± 0.07	−3.47 ± 0.05	−3.41 ± 0.02	−3.41 ± 0.02
Intercept	40.05 ± 0.07	39.92 ± 0.28	40.29 ± 0.22	40.17 ± 0.12	40.23 ± 0.09
Efficiency	97.11	96.44	93.75	96.57	95.73
*R* ^2^	1.000	0.998	0.999	0.999	1.000
Pooled slope = −3.42 (*p* = 0.66). Pooled intercept = 40.14 (p = 0.08)					

Slope and intercept values are shown as mean ± SD. All assays included non-template controls that never produced a positive amplification. See also Figure 2A.

**Table 4:** Cell-based standard curves for *Apocalathium malmogiense*, *Biecheleria baltica*, and *Gymnodinium corollarium* generated on separate occasions (*n* = 4).

	Assay 1	Assay 2	Assay 3	Assay 4
*Apocalathium malmogiense*, SHTV-5				
Slope	−3.47 ± 0.03	−3.49 ± 0.03	−3.47 ± 0.01	−3.45 ± 0.02
Intercept	33.50 ± 0.09	33.62 ± 0.09	33.79 ± 0.04	34.03 ± 0.07
Efficiency	94.21	93.43	94.21	94.96
*R* ^2^	0.999	0.999	0.999	0.999
*Biecheleria baltica*, WHTV-S1				
Slope	−3.53 ± 0.02	−3.38 ± 0.03	−3.45 ± 0.01	−3.58 ± 0.02
Intercept	35.59 ± 0.07	35.66 ± 0.09	35.86 ± 0.03	35.23 ± 0.07
Efficiency	91.99	97.67	94.92	90.25
*R* ^2^	0.999	0.999	1.000	0.999
*Gymnodinium corollarium*, GCTV-B4				
Slope	−3.44 ± 0.03	−3.65 ± 0.07	−3.48 ± 0.07	−3.53 ± 0.02
Intercept	35.87 ± 0.10	36.61 ± 0.22	36.31 ± 0.21	36.58 ± 0.07
Efficiency	95.30	87.92	93.84	91.85
*R* ^2^	0.999	0.998	0.998	0.999

Slope and intercept values are shown as mean ± SD. All assays included non-template controls that never produced a positive amplification. See also Figure 2B.

**Table 5:** Reproducibility of the qPCR assays based on standard curves with synthetic DNA dilutions (*n* = 5).

Species/strain	Gene copies in the reaction	Mean Ct ± SD	CVCt (%)
*Apocalathium malmogiense* SHTV-5	7.5 × 10^6^	17.26 ± 0.17	1.02
	7.5 × 10^5^	20.79 ± 0.10	0.51
	7.5 × 10^4^	24.17 ± 0.15	0.65
	7.5 × 10^3^	27.67 ± 0.08	0.33
	7.5 × 10^2^	31.04 ± 0.36	1.16
	7.5 × 10^1^	34.57 ± 0.37	1.07
*Biecheleria baltica* WHTV-S1	7.5 × 10^6^	16.62 ± 0.27	1.60
	7.5 × 10^5^	20.03 ± 0.16	0.76
	7.5 × 10^4^	23.49 ± 0.22	0.94
	7.5 × 10^3^	26.92 ± 0.24	0.88
	7.5 × 10^2^	30.71 ± 0.30	0.98
	7.5 × 10^1^	34.73 ± 0.87	2.50
*Gymnodinium corollarium* GCTV-B4	7.5 × 10^6^	16.58 ± 0.19	1.12
	7.5 × 10^5^	20.01 ± 0.11	0.55
	7.5 × 10^4^	23.4 ± 0.14	0.62
	7.5 × 10^3^	26.96 ± 0.14	0.54
	7.5 × 10^2^	30.46 ± 0.28	0.93
	7.5 × 10^1^	33.60 ± 0.19	0.56

**Table 6:** Reproducibility of the qPCR assays based on standard curves with algal DNA dilutions (*n* = 4).

Species/strain	Cell number in the reaction	Mean Ct ± SD	CVCt (%)
*Apocalathium malmogiense* SHTV-5	8 × 10^4^	16.83 ± 0.21	1.26
	8 × 10^3^	20.16 ± 0.27	1.37
	8 × 10^2^	23.68 ± 0.36	1.53
	8 × 10^1^	27.07 ± 0.29	1.10
	8 × 10^0^	30.68 ± 0.28	0.93
	8 × 10^−1^	34.12 ± 0.12	0.37
*Biecheleria baltica* WHTV-S1	5 × 10^4^	19.20 ± 0.62	3.26
	5 × 10^3^	22.62 ± 0.48	2.16
	5 × 10^2^	26.12 ± 0.55	2.11
	5 × 10^1^	29.61 ± 0.37	1.28
	5 × 10^0^	33.08 ± 0.41	1.27
	5 × 10^−1^	36.61 ± 0.21	0.58
*Gymnodinium corollarium* GCTV-B4	9 × 10^4^	18.94 ± 0.17	0.91
	9 × 10^3^	22.44 ± 0.37	1.67
	9 × 10^2^	25.73 ± 0.18	0.71
	9 × 10^1^	29.20 ± 0.29	1.02
	9 × 10^0^	33.05 ± 0.25	0.78
	9 × 10^−1^	36.57 ± 0.42	1.16

#### 3.2.1 Gene-based standard curves

**Figure 3:**
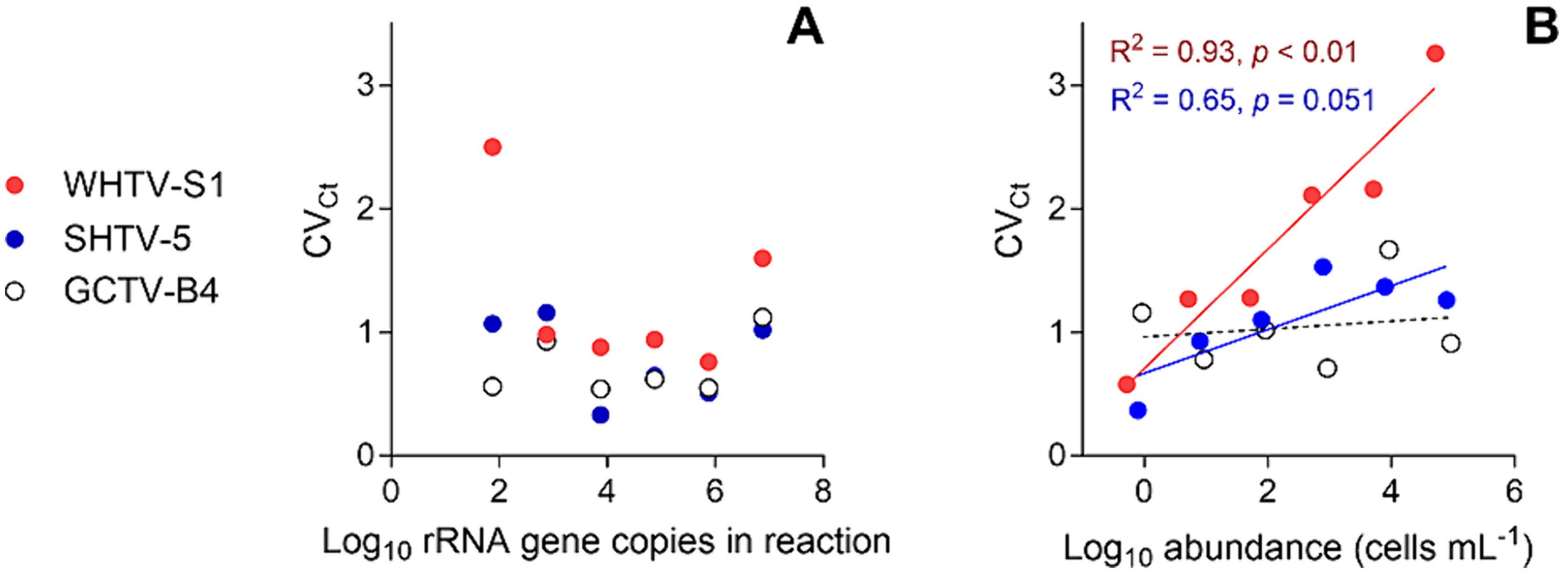
Inter-assay variation assessment for the target species and strains: *Apocalathium malmogiense* (SHTV-5), *Biecheleria baltica* (WHTV-S1), and *Gymnodinium corollarium* (GCTV-B4). Relationships between the CV_Ct_ values and **(A)** rRNA gene copy number in the reaction (*n* = 5) and **(B)** cell abundance in the cell-based qPCR assays (*n* = 5). Significant regressions for cell-based assays with WHTV-S1 and SHTV-5 are shown in the coding color. See Tables 3, 4 for the primary data for regression analysis.

The gene-based standard curves covered linear detection over six orders of magnitude, with a mean qPCR efficiency of 96, 94 and 91% for *G. corollarium*, *A. malmogiense*, and *B. baltica*, respectively (Table 3 and Figure 2A). The detection limit tested was 75 gene copy numbers in all assays. The Ct for the lowest gene copy number tested varied from 33.6 in GCTV to 34.7 in WHTV, so it is not likely that the sensitivity lower than 75 gene copy numbers can be achieved. The inter-assay variation was low, with CV_Ct_ mean values of the standard curves being 1.3, 0.8 and 0.7% for *B. baltica*, *A. malmogiense*, and *G. corollarium*, respectively (Table 5 and Figure 3A). The CV_Ct_ was not related to the copy number in the reaction in all assays. The pooled slope and intercept, calculated for the respective target species standard curve, are provided in Table 3.

#### 3.2.2 Cell-based standard curves

The cell-based standard curves covered linear detection over six orders of magnitude, with a mean qPCR efficiency of 94% for *A. malmogiense* and *B. baltica*, and 92% for *G. corollarium* (Table 4 and Figure 2B). For all three species, the detection limit tested was less than one cell in the reaction. The CV_Ct_ mean values of the standard curves for the target species were 1.7, 1.1 and 1.0% for *B. baltica*, *A. malmogiense* and *G. corollarium*, respectively, and remained at ≤1.0% for all species in the low cell abundance range (1 to 10^2^ cells mL^−1^). In *B. baltica*, the CV_Ct_ was significantly positively related to the cell abundance, whereas the relationship for *A. malmogiense* was nearly significant (Figure 3). In *G. corollarium*, the CV_Ct_ was the lowest among the three species (Table 6) and unrelated to the cell abundance (Figure 3).

#### 3.2.3 Evaluation of primer-probe specificity

No cross-reactivity for any of the laboratory strains of *A. malmogiense*, *B. baltica* and *G. corollarium* was observed (Table 7). Similarly, no amplification was observed for DNA extracted from the cultures of *Heterocapsa triquetra*, *Peridiniella catenata*, and *Protodinium simplex*, except for a few cases where a non-specific product was observed after more than 35 cycles (i.e., beyond the dynamic range of the assays; Figure 2A). Sequencing of the qPCR-products from the spring bloom field sample DNA confirmed the presence of all three target species and the specificity of the assays. Moreover, when the environmental DNA extracted from the field sample collected in September 2010 was used as a template, no specific amplification was observed with any of the primer-probe sets selected for the species-specific qPCR assays for *A. malmogiense*, *B. baltica* and *G. corollarium*, which was consistent with the absence of these species in the phytoplankton community at the time. However, for the *B. baltica* and *G. corollarium* primer-probe sets, some unspecific amplification occurred at Ct >34, corresponding to the end of the standard curve and outside of the standard curve, respectively.

**Table 7:** Cross-reactivity of the qPCR primer-probe sets for ecologically and phylogenetically relevant dinoflagellates included in the battery of test species/strains; see Table 1 for details.

Species (strain), 10 X dilution of DNA	Primer-probe set for *A. malmogiense*	Primer-probe set for *B. baltica*	Primer-probe set for *G. corollarium*
*A. malmogiense* (SHTV)	+	−	−
*A. malmogiense* (SHTV-0910)	+	−	−
*A. malmogiense* (SHTV-0908)	+	−	−
*A. malmogiense* (SHTV-5)	+	−	−
*B. baltica* (WHTV)	−	+	−
*B. baltica* (WHTV-15)	−	+	−
*B. baltica* (WHTV-S8)	−	+	−
*B. baltica* (WHTV-S1)	−	+	−
*G. corollarium* (GCTV-B4)	−	−	+
*G. corollarium* (GCTV-03)	−	−	+
*G. corollarium* (GCTV-A3)	−	−	+
*H. triquetra* (HTF1002)	+ at Ct >37	−	−
*P. catenata* (PCTV-0907)	−	−	+ at Ct >37
*P. simplex* (K-0661)	−	−	−
Field material DNA (station H4, fall)	−	+ at Ct >34	+ at Ct >34

For non-specific amplifications, the Ct values are provided.

#### 3.2.4 Correspondence between microscopy-and qPCR-based estimates of cell abundance

**Figure 4:**
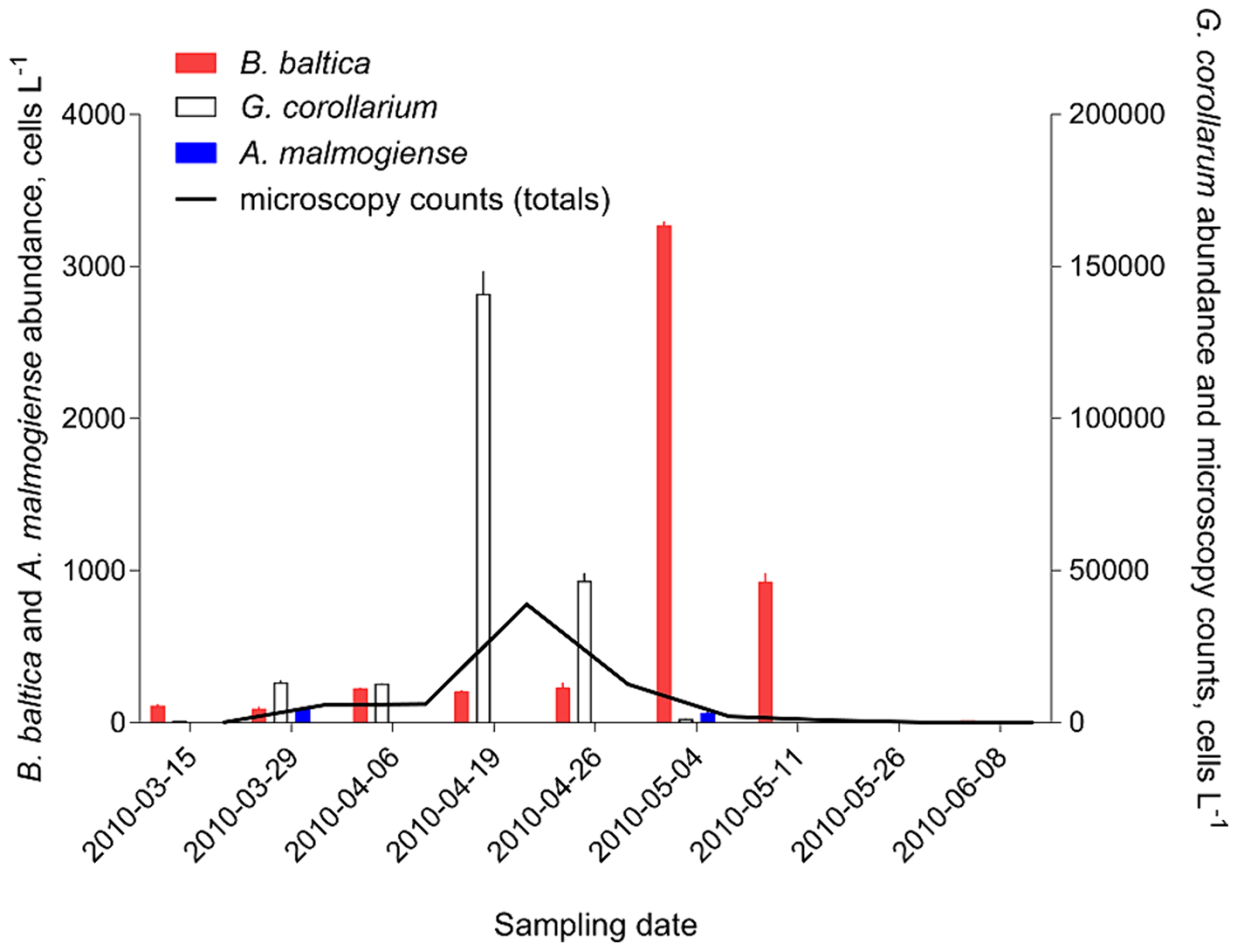
Correspondence for cell abundance estimates between microscopy-and qPCR-based assays obtained for the test strains of the laboratory cultures of *Apocalathium malmogiense* (SHTV-5), *Biecheleria baltica* (WHTV-S1) and *Gymnodinium corollarium* (GCTV-B4). Horizontal and vertical error bars indicate 95% confidence interval based on three replicate measurements; note that in most cases, this variability is not visible. See Figure 5 for the strain-specific values of the rRNA gene copy number used in the calculations of cell abundance in the qPCR-based estimates and Table 8 for statistical details on the linear regression for each species.

There was a very strong (*R*^2^ > 0.98 in all cases) relationship between the cell abundance estimates based on qPCR and the Utermöhl counts (Table 8). However, the qPCR assays consistently overestimated the number of cells in the culture samples used in the laboratory testing (Figure 4). Moreover, neither the slopes nor the intercepts of the regression lines for the test strains were significantly different (*slopes*: *F*_2,12_ = 2.597, *p* > 0.12; the pooled slope equals 5.12; *intercepts*: *F*_2,14_ = 0.059, *p* > 0.94; the pooled intercept equals −217).

**Table 8:** Regressions for cell abundance estimates derived from the microscopy counts and qPCR assays for *Apocalathium malmogiense*, *Biecheleria baltica*, and *Gymnodinium corollarium*.

Regression parameters	*A. malmogiense*	*B. baltica*	*G. corollarium*	Pooled regression
Slope	5.66 ± 0.47	4.47 ± 0.32	5.13 ± 0.22	5.12
Intercept	−1,230 ± 1,721	616.0 ± 1,007	−131 ± 900	−217
*R* ^2^	0.9728	0.9798	0.9927	0.999
*p-*value	0.0003	0.0002	<0.0001	<0.0001
Comparison of slopes and intercepts (*F*-test) for all species		*Slopes*: *F*_2,12_ = 2.596; *p* > 0.12; *Intercepts*: *F*_2,14_ = 0.058; *p* > 0.9		

Slope and intercept values are shown as mean ± SD; *n* = 3. See also Figure 4.

#### 3.2.5 Screening environmental samples for target species

**Figure 5:**
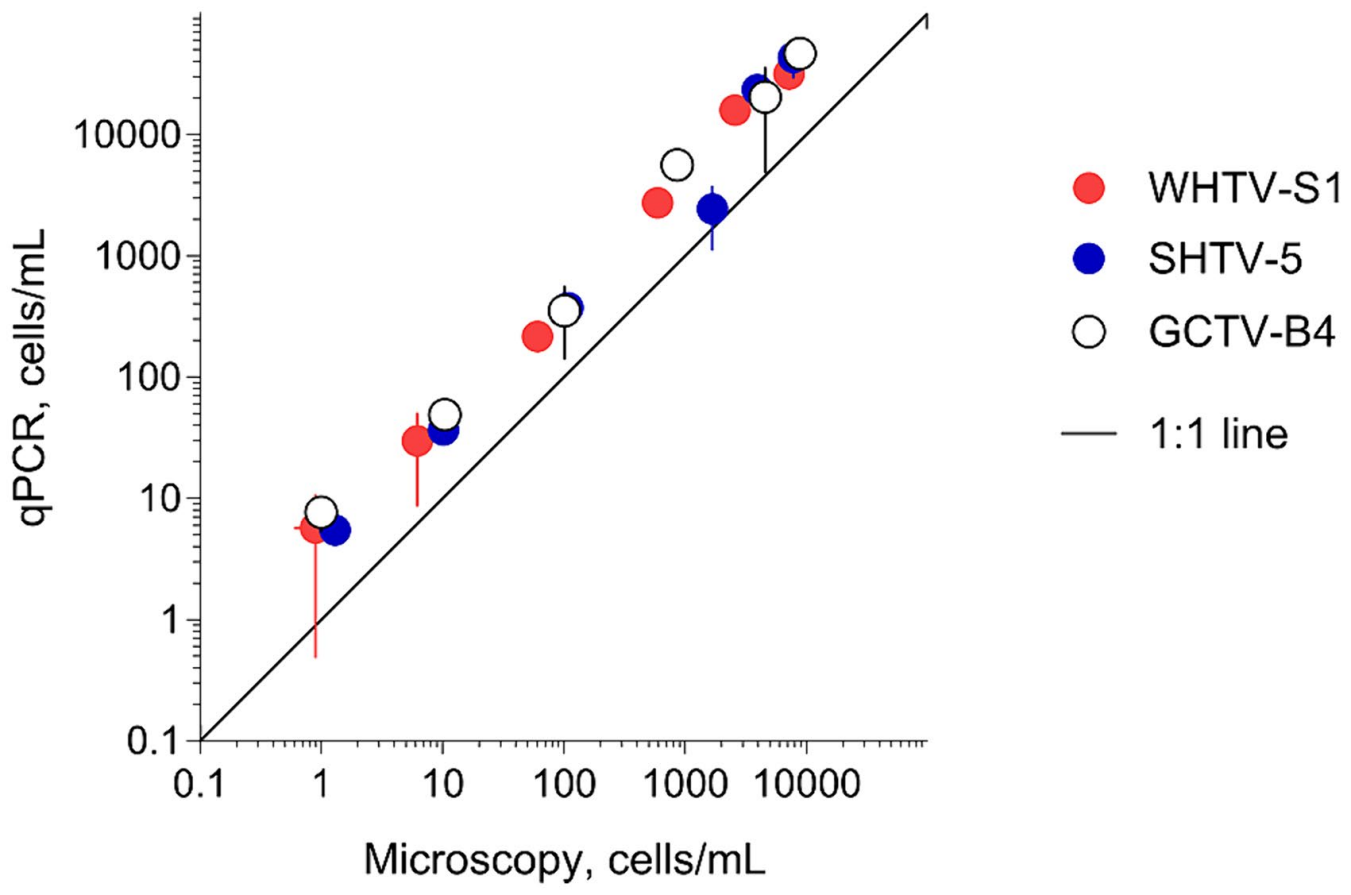
Variation in rRNA gene copy number per cell in the species and strains tested (Table 1). For each strain, two replicate samples were tested with three technical replicates. Mean value for all strains within a species is indicated below the species name and mean value for the strains used for the assay development (WHTV-S1, SHTV-5, and GCTV-B4) is indicated above the data points for these strains.

**Figure 6:**
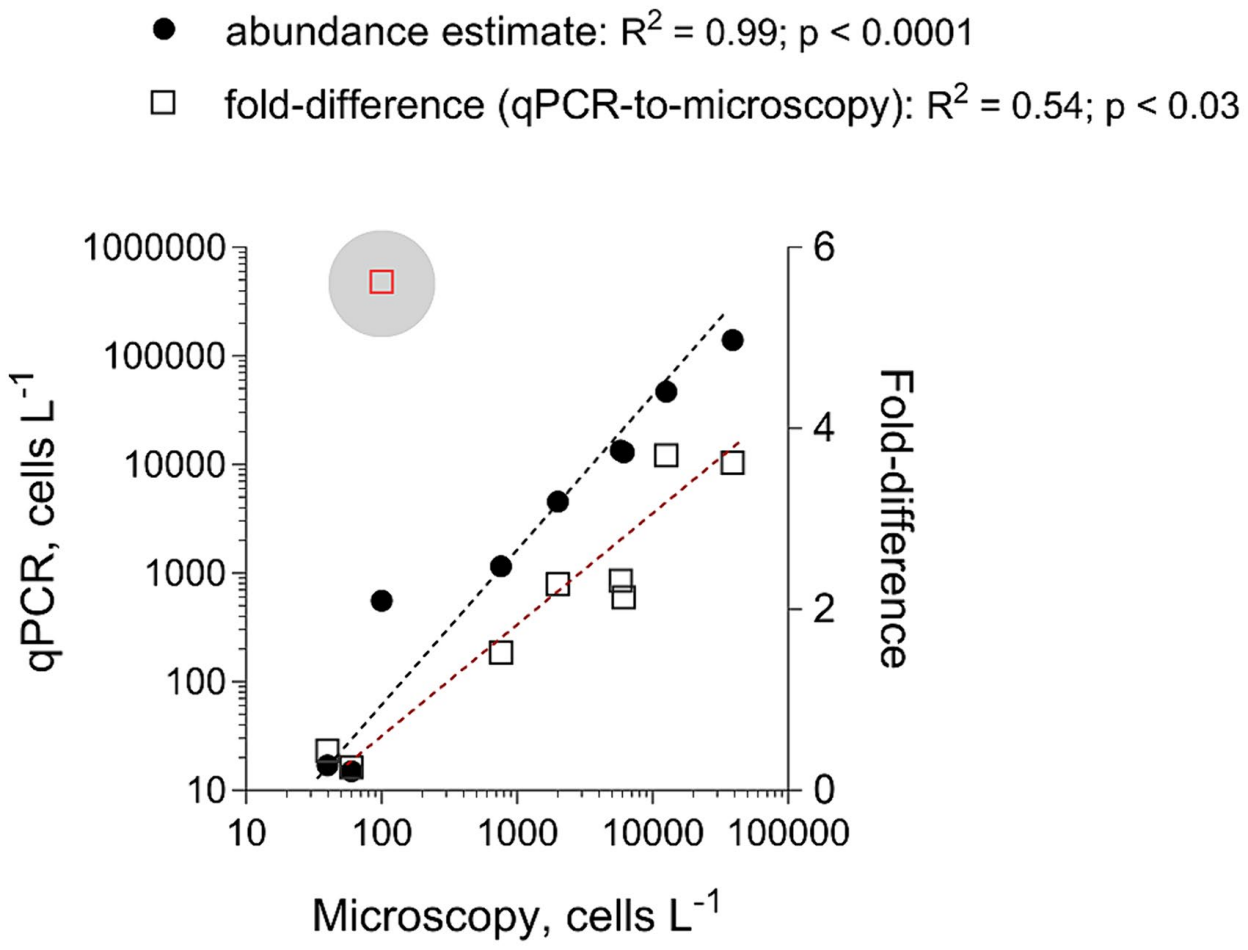
Application of the developed qPCR assays to field screening for the target species in the plankton samples collected in the Himmerfjärden Bay (H4) during the spring bloom period in 2010. For each species, a sample collected on each occasion was analyzed in triplicate using a respective synthetic standard and applying an average gene copy number determined for this species (Figure 5). The same samples were analyzed by light microscopy and the data for the entire species complex.

**Figure 7:**
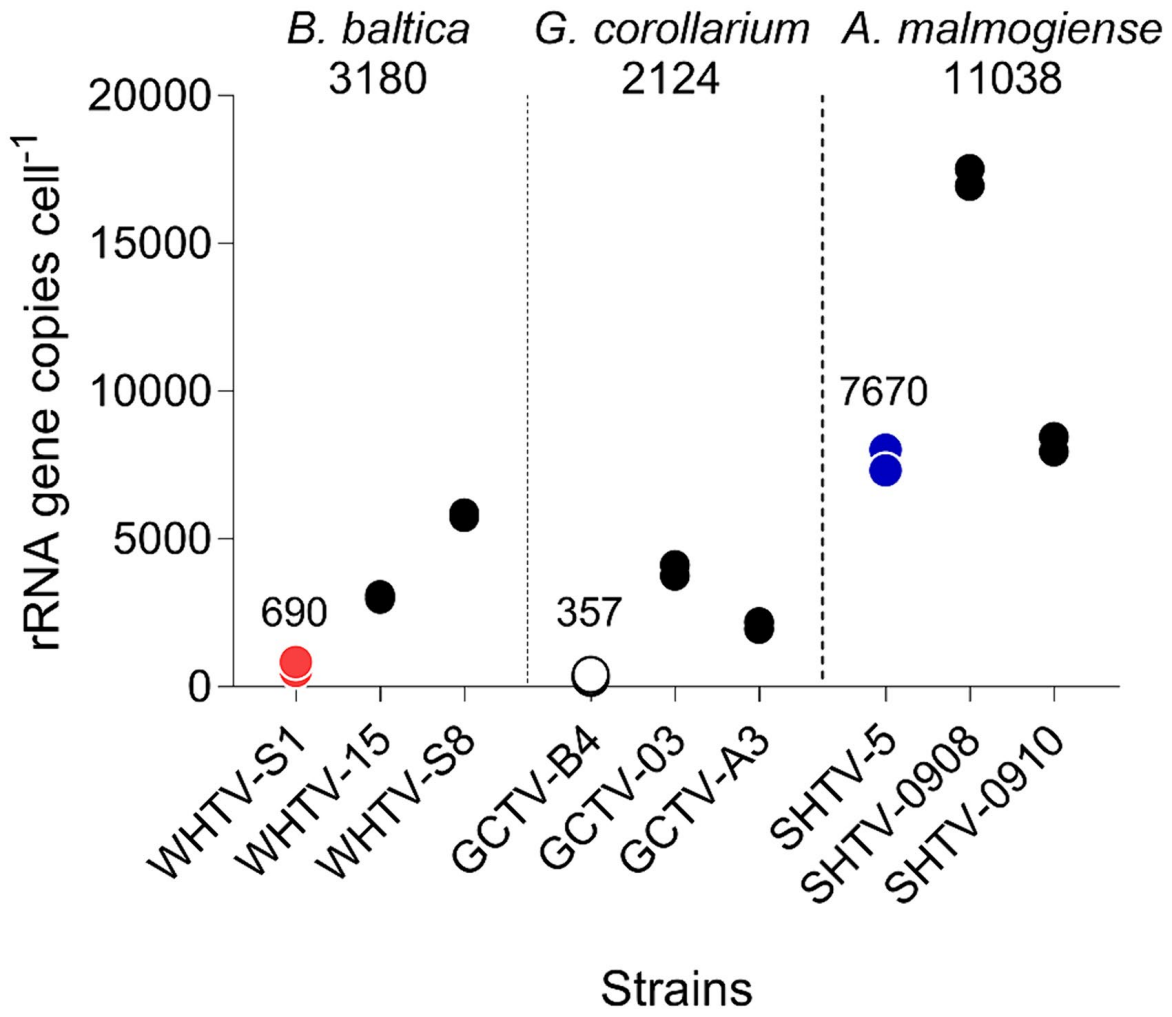
Correspondence for the total abundance (cells L^−1^) of the dinoflagellates belonging to *Apocalathium*/*Biecheleria*/*Gymnodinium* complex between estimates derived from the microscopy counts (no species-level identification is possible) and the qPCR assays targeting each species. The regression for the qPCR vs. microscopy was based on all data points, whereas the extreme value of 5.4 (indicated with a blue circle) was excluded from the regression for the fold-difference as a function of microscopy counts. See Figure 6 for the primary data.

To evaluate the adequacy of the developed qPCR assays for environmental screening, they were applied to environmental community DNA extracts from plankton collected in a coastal area of the Northern Baltic Proper during spring bloom 2010 (Figure 1). For consistency, the pooled slope and intercept for the plasmid standard curve for each species (Table 3) were used when estimating cell abundances in the field samples. To convert the Ct values to the cell abundance estimates, we applied the average rRNA gene copy number observed for each species (Figure 5). Variable cell abundance was observed for the dinoflagellates comprising the *Apocalathium*/*Biecheleria*/*Gymnodinium* species complex, with clear peaks for *G. corollarium* and *B. baltica* (Figure 6). The most common species was *G. corollarum* detected at high abundance on all sampling occasions, whereas *A. malmogiense* was detected at low abundances and only twice, and moderate quantities of *B. baltica* were detected on all sampling occasions. The regression for the qPCR vs. cell counts was highly significant, although the qPCR-based estimates in most cases exceeded those based on the light microscopy (2.4-fold on average; Figure 7). Within the observed range of the dinoflagellate abundance (4 to 4 × 10^5^ cells L^−1^), there was a significant positive relationship between the fold difference (between the qPCR and cell counts) and the total dinoflagellate abundance when the extreme value of 5.4-fold difference observed on the 15th of March was excluded from the regression.

## 4 Discussion

Application of taxa-specific qPCR assays for the *Apocalathium*/*Biecheleria*/*Gymnodinium* complex could substantially enhance our understanding of hidden plankton biodiversity, seasonal dynamics, and trophic interactions. Here, we developed qPCR assays that can be used to rapidly detect co-occurring and morphologically similar dinoflagellates in plankton samples collected in the Baltic Sea. Species-specific qPCR primers and probes with high specificity and sensitivity were developed for three species, *A. malmogiense*, *B. baltica*, and *G. corollarium*. We also established that these primer-probe sets were effective in measuring the abundance of all three species in a coastal area of the Northern Baltic Proper.

The cross-reactivity of the primers designed in this study showed high specificity for each target species while not amplifying when tested against other dinoflagellates reported from the study area. The species tested for cross-reactivity were chosen because they represented species that are genetically most similar to each target species for the ITS region; therefore, it is likely that the most likely candidates for false identification were included in the testing. Notably, the forward primer for *Apocalathium malmogiense*, S459F, also matches the ITS sequence of *A. aciculiferum* (Lemmermann) Craveiro, Daugbjerg, Moestrup & Calado (Craveiro et al., 2017). The reason is that *A. malmogiense* and *A. aciculiferum* have identical rDNA sequences and are believed to have diverged very recently (Logares et al., 2007). Therefore, it is likely that a positive amplification for *A. aciculiferum* can be obtained using the primer-probe system developed for *A. malmogiense*, but we have not tested it. However, besides having different morphological features compared to *A. malmogiense*, *A. aciculiferum* is a freshwater species inhabiting lakes, and it has not been reported in the marine environments, including the Baltic Sea. Therefore, we believe that the selected system would be specific for *A. malmogiense* in the Baltic plankton communities.

The mean amplification efficiencies for the developed gene-and cell-based assays targeting *G. corollarium*, *A. malmogiense* and *B. baltica* were >90%, with a 6-log linear dynamic range of quantification (Tables 3, 4). The assays were sensitive as indicated by the fact that less than a single cell or 75 gene copies per reaction were sufficient to obtain a positive amplification. These quantification limits (Tables 5, 6) suggest the high rRNA gene copy number per cell (~75 copies/cell on average). Indeed, as in other dinoflagellates, the rDNA operon is tandemly repeated up to thousands of copies (Saito et al., 2002; Galluzzi et al., 2004), which is supported by the observed variability in the rRNA gene copy numbers within and across the species (Figure 5). It is also possible that within-strain variability is also substantial depending on the life cycle and growth conditions, as reported for several *Alexandrium* species in the Mediterranean Sea (Galluzzi et al., 2010).

Absolute quantification is usually based on standard curves for target DNA using cell-based assays. However, the use of such assays for dinoflagellate quantification is complicated by the high and variable rRNA gene cell copy number (Guo et al., 2016). As we had no prior knowledge of the copy number variability between the target species and between different isolates of the same species, we also used the synthetic gene standard to determine the relative difference in rDNA copy numbers across our target species and the test strains (Figure 5). We found that there are indeed differences between the copy numbers both between the species and between the isolates within a single species, with the highest copy number observed in *A. malmogiense* and the lowest in *G. corollarium*. Moreover, the copy numbers varied by 8-, 11-and 2-fold, between different isolates of *B. baltica*, *G. corollarium*, and *A. malmogiense*, respectively. The rRNA cell copy numbers determined in this study were within the range reported for other dinoflagellates (Galluzzi et al., 2004, 2010; Penna and Galluzzi, 2013).

Although all species listed in Table 1 were included in the specificity testing, cell copy numbers were only investigated for the three target species, and the variability observed for these test species and strains suggests that including more cultures in the cell copy number comparisons would increase the span of the observed values. Clearly, the use of a cell-based standard curve would require more understanding of the ecological and evolutionary drivers of the rDNA operon variability for the species in question. To address these challenges, the synthetic gene approach is very instrumental and can be used for screening field samples and determination of the absolute abundance of the target rRNA gene as well as the determination of cell copy number in specific strains and isolates. Given the observed cell copy numbers (Figure 5) and the dynamic range of the assays (Tables 5, 6), the sensitivity of the gene-based assays allows the analysis of less than a single cell in a qPCR reaction. Such high sensitivity ensures accurate detection and quantification, even in complex, mixed-species samples at ecologically relevant abundances.

Accuracy of the absolute quantification approach relies on the quality of standard curve construction by controlling the precision and reproducibility (Rutledge and Côté, 2003). Here, for all three species, the linearity was excellent, with *R*^2^ values greater than 0.99. A standard curve is generally considered high quality if the correlation between the log-copy numbers and the Ct values is to 0.99 or higher. However, the regression coefficient alone does not fully reflect the precision or accuracy achieved (Rutledge and Côté, 2003). Comparing Ct values across five independent runs of the cell-based assays, we found an average CV_Ct_ of less than 2% for all species (Table 6 and Figure 3B), indicating good reproducibility between runs. Moreover, in the low range of the target cells, the CV_Ct_ values were below 1% (Table 3), indicating that at the environmentally relevant abundance of the dinoflagellates, the assays are highly reproducible. As expected, the inter-assay variation for the gene-based assays was even lower (CV_Ct_ <1%; Table 5 and Figure 3A), emphasizing the usefulness of the synthetic standard for internal quality control of the assay reproducibility (Vermeulen et al., 2009; Conte et al., 2018).

A correspondence was found between the results obtained with microscopy and those found with each of the qPCR assays applied to the single-species cultures (Figure 4). This high correlation was also evident when the abundance of the entire *Apocalathium*/*Biecheleria*/*Gymnodinium* complex was estimated during the bloom event in a coastal area of the Northern Baltic Proper (Figures 6, 7). However, the qPCR assays consistently overestimated the cell abundance, both in the laboratory tests with monocultures (5-fold; Figure 4) and in the mixed field-sampled plankton (2-fold; Figure 7). A difference of this magnitude would lead to considerably different abundance estimates of the target species. Moreover, the discrepancy between the qPCR-based estimates and the microscopy counts increased significantly with increasing cell abundance, indicating that during the peak bloom, the uncertainty would increase. It is well-established that qPCR assays can overestimate cell numbers compared to microscopy counts (Murray et al., 2019; Kim et al., 2024) due to several factors. Variability in gene copy number per cell, presence of extracellular DNA, and differences in DNA extraction efficiency can all contribute to higher DNA yields. Additionally, qPCR can detect DNA from cells that are decaying and, therefore, are difficult to count microscopically. For this study, the variable gene copy number and extracellular DNA are likely the most influential factors. The mounting evidence of intra-strain variability in detectable rDNA copy numbers as a function of their growth rate could have severe implications for qPCR-based cell enumeration of dinoflagellates, but also other algae, such as diatoms (Guo et al., 2016), and requires further investigation.

In conclusion, this study demonstrates the usefulness of real-time PCR as a sensitive and rapid molecular technique for the detection and quantification of *A. malmogiense*, *B. baltica*, and *G. corollarium* from environmental samples. For each species, the inter-assay variation of the cell-based assays was low, with CV_Ct_ <1% in the ecologically relevant range of population abundance, which facilitates their applicability in the analysis of the monitoring or other samples that are collected and analyzed continuously. The assays developed were highly specific and sensitive in the unambiguous detection of all three species, and thus are valuable for routine plankton, biogeographic and phylogenetic investigations. Future studies should address ecological and phylogenetic aspects of rRNA copy number variability and improve its assessment in the monitoring and field studies of *A. malmogiense*, *B. baltica*, and *G. corollarium*.

## Data Availability

The datasets presented in this study can be found in online repositories. The names of the repository/repositories and accession number(s) can be found in the article/Supplementary material: see the tables and Section 2 “Materials and methods”, as well as Zenodo repository 10.5281/zenodo.13762469.

## Footnotes

^1^http://www.ncbi.nlm.nih.gov/BLAST
