# Supplementary material for: Quantitative real-time PCR assays for species-specific detection and quantification of Baltic Sea spring bloom dinoflagellates
Source: Front Microbiol. 2024 Sep 24;15:1421101. doi: 10.3389/fmicb.2024.1421101 (PMC11458424; doi:10.3389/fmicb.2024.1421101)
Supplement: Supplementary file 1 [file Data_Sheet_1.PDF]

Supplementary Material

Supplementary Text 1

Methodology background

Quantitative polymerase chain reaction (qPCR) assays targeting the ribosomal RNA (rRNA) encoding gene (rRNA operon) have become a popular tool for species identification and quantification in environmental samples of dinoflagellates (Bowers et al. 2000; Galluzzi et al. 2004; Dyhrman et al. 2006), particularly toxin-producing species with high capacity for outbreaks (Toebe et al. 2013; Smith et al. 2014; Kon et al. 2015; Hernández-Becerril et al. 2018; Engesmo et al. 2018; Ruvindy et al. 2018). In addition to high taxonomic resolution, these assays also have a sensitivity surpassing that of the microscopy-based techniques. For example, an assay developed for *Gymnodinium catenatum* estimated cell densities in environmental samples as low as 0.07 cells per PCR reaction or three cells per liter and detected the presence of cells below the detection limit of light microscopy (Toebe et al. 2013; Smith et al. 2014). Several types of real-time PCR assays with differing levels of specificity have been developed, and positive reactions are detected either with a fluorescent reporter probe (e.g., hydrolysis probes, molecular beacons, locked-nucleic acid bases [LNA]) or a double-stranded DNA-binding dye (e.g., SYBR green). As a target region for distinguishing dinoflagellate species, Internal Transcribed Spacer (ITS1 and ITS2) of the rRNA operon has been recommended as a high-resolution marker (Stern et al. 2012).

Supplementary Figure 1. An example of pMA-T plasmid map for synthetic gene used for qPCR as a standard. Here, the construct corresponding to the target ITS1 region of *Biecheleria baltica,* strain WHTV, is shown. The plasmid maps for the other two target species were constructed in the same way using *SfiI* and *SfiI* cloning sites.

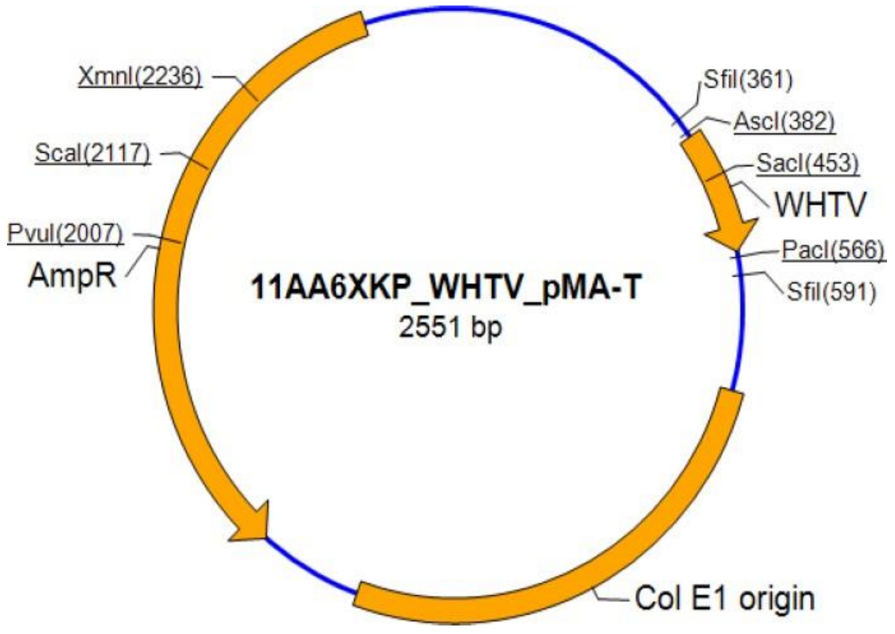

Supplementary Table 1. Primers and probes designed in this study for the target species and the expected product size. Primers/probe pairs selected for the qPCR testing are indicated in Italics; those that were superior in these tests and used in the analysis are in bold.

Species	Primer/Probe	Sequence (5'→ 3')	Region	Product size (bp)						
Apocalathium malmogiense	*Forward primer S366F*	*CTGCTTCAGTGTCCAATCCAT*	*ITS2*				*139*	177		
	Forward primer S394F	*CGGCGACATAATCCACATCT*	*ITS2*						*144*	*149*
	Forward primer S416F	*GGTGTTGTTGTCTGAGTGTGG*	*ITS2*		79	*89*				
	Forward primer S459F	***GTCCTTGACGCATTCAGAGC***	***ITS2***	***84***						
	Reverse primer S494R	CAGATGAAATCCCCATGCTCT	ITS2		79					
	Reverse primer S504R	*GTTGTGCGACCAGATGAAATC*	*ITS2*			*89*	*139*			
	Reverse primer S537R	*GCACCAACAAGCAACATCAG*	*ITS2*						*144*	
	Reverse primer S542R	***GACATGCACCAACAAGCAAC***	***ITS2***	***84***				177		*149*
	Probe S442	*6FAM-TGTTAAGGTGCTCTCGGGTC-MGBNFQ*	*ITS2*		79	*89*	*139*	177		
	Probe S484	***6FAM-GATTTCATCTGGTCGCACAA-MGBNFQ***	***ITS2***	***84***					*144*	*149*
Biecheleria baltica	Forward primer B49F	CTATGTGAGTGACTGGGTGGAG	ITS1		163					
	Forward primer B51F	***ATGTGAGTGACTGGGTGGAGA***	***ITS1***	***161***						
	Forward primer B86F	GCGCACTTCCTCCATGTG	ITS1			126				
	Forward primer B134F	ACGCGTGTCGTTCCTTGT	ITS1				*78*			
	Forward primer B504F	GCAGGAGTGTGAAGAGAGCTG	ITS2					98	104	
	Reverse primer B211R	***GACAGTTGAGCCAGAGCAAAC***	***ITS1***	***161***	163	126	*78*			
	Reverse primer B601R	GCATGGGAGAGTTGTTGGTT	ITS2					98		
	Reverse primer B607R	TGCTGTGCATGGGAGAGTT	ITS2						104	
	Probe B169	***6FAM-AGGTTGTTCCTGTTGCCATT-MGBNFQ***	***ITS1***	***161***	163	126	*78*			
	Probe B562	6FAM-ATAGATTCTTGCAGCGCTCC-MGBNFQ	ITS2					98	104	
Gymnodinium corollarium	Forward primer G438F	GAGTGCTCGTGCCTCAAGAT	ITS2		*165*					
	Forward primer G440F	GTGCTCGTGCCTCAAGATG	ITS2				*163*			
	Forward primer G452F	CAAGATGCGCAGTGTCTACC	ITS2			151				
	Forward primer G458F	***GCGCAGTGTCTACCTTCGT***	***ITS2***	***145***						
	Reverse primer G602R	***CAGAGCCGTGAGAAGACAGG***	***ITS2***	***145***		151	*163*			
	Reverse primer G604R	CACAGAGCCGTGAGAAGACA	ITS2		*165*					
	Probe G535	***6FAM-TACTGAGCATCTCGGTGTGC-MGBNFQ***	***ITS2***	***145***	*165*	151	*163*			

Supplementary Text 2

Pilot tests: Optimization and comparison of DNA extraction methods

Three methodological aspects were evaluated: (I) DNA extraction efficiency in Lugol-preserved material, (II) cell recovery efficiency from the filter, and (III) selection of medium for harvesting algal cells from the filter. These pilot tests were needed to optimize the extraction method to ensure effective DNA recovery and quality, particularly for Lugol-preserved material due to the challenges posed by this preservation method (Eckford-Soper and Daugbjerg, 2015). Moreover, when following the conventional procedure for DNA extraction for filter-collected phytoplankton, including dinoflagellates (Penna et al., 2006; Penna and Galluzzi, 2013), we observed that many cells did not detach from the filter when washed and resuspended in the lysis solution. This prompted us to investigate the extent of DNA loss when the filter was removed. Finally, we tested two alternative media to improve the cell harvesting from the filter.

I. DNA extraction

Methods. The extractions of DNA from fresh and Lugol-preserved cultures (*Gymnodinium corollarum*, strain GCTV-B4) and field-collected mixed plankton samples were compared among three commercially available methods: Qiagen DNeasy Plant Mini Kit (Protocol 1), DNAreleasy (Protocol 2), and Chelex (Protocol 3); see **Supplementary Table 2**. For all extractions, 15 mL of fresh and Lugol-preserved GCTV-B4 culture or 25 mL of plankton sample (pre-filtered with 90-µm sieve) were centrifuged for 10 minutes at 5,000 rpm. The Lugol-preserved sample was washed once with artificial seawater and centrifuged again. The resulting pellet was transferred to a 2 mL Eppendorf tube. This involved two additional centrifugations: the first to rinse the 15 mL tube and transfer all material to the Eppendorf tube, and the second to remove the supernatant before freezing at -80°C.

Samples were homogenized with glass beads using a FastPrep®-24 Instrument. The DNA was extracted following the manufacturer guidelines for Protocols 1 and 2. For Protocol 3, Chelex® 100 solution (200 µL) was added to the samples, followed by heating at 50°C for 30 minutes and then at 105°C for 8 minutes (Motwani and Gorokhova, 2013). After centrifugation, the DNA-containing supernatant was collected and stored at 8°C overnight. For full details, see Section 2.4.1 of the paper.

When comparing DNA quantity and quality across the methods, the primary criterion was ensuring that the results from Lugol-preserved samples closely matched those from fresh material, as routine monitoring samples are preserved in Lugol. Therefore, it was essential for the method to be applicable to the regular monitoring samples. The DNA yield and method simplicity were also important, whereas DNA quantity was considered acceptable if qPCR efficiency was sufficiently high.

The DNA concentration in each sample was quantified fluorometrically by staining with Hoechst dye 33258 (Sigma-Aldrich), and the DNA quality was assessed using absorbance ratio at 260/280 and 230/260 nm using Nanophotometer (Implen). The two methods that provided the best results were then used for qPCR with GCTV-B4, and their reaction efficiencies were compared to select the most effective one.

Results. The outcome of the comparisons is presented in **Supplementary Figure 2**. The discrepancy between the Lugol-preserved and fresh samples for each type of material (*Gymnodinium* strain and wild plankton) was evaluated as the Euclidean distance for the DNA yield. The Chelex method (Protocol 3) provided the lowest discrepancy between the Lugol-preserved material, and the highest DNA quantity for the preserved samples, yet the lowest DNA quality (**Supplementary Table 2)**, whereas the two other kits provided consistently higher yields for the fresh samples yet much lower yields for the Lugol-preserved ones. However, DNA purity was higher for the DNeasy Plant Mini Kit compared to the other two protocols (**Supplementary Table 2**).

The two protocols with the most promising outcome were Chelex (best correspondence between the preservation methods) and DNeasy Plant Mini Kit (best DNA quality). Therefore, they further evaluated using qPCR, where Chelex showed the higher reaction efficiency (Chelex: $R^2 = 1$, Efficiency = 96.234; DNeasy Plant Mini Kit: $R^2 = 0.998$, Efficiency = 91.415), making it the preferred method for our downstream applications.

Conclusion. Among the three protocols, the Chelex method provided the least deviation between Lugol-preserved and fresh samples and the best balance of efficiency, simplicity, and DNA quality (Protocol 1). Moreover, in the qPCR evaluation, Chelex showed the highest reaction efficiency, making it the preferred method for downstream applications. Also, the Chelex method involved fewer steps, reducing variability between samples, and thus, it was quick, simple and reliable.

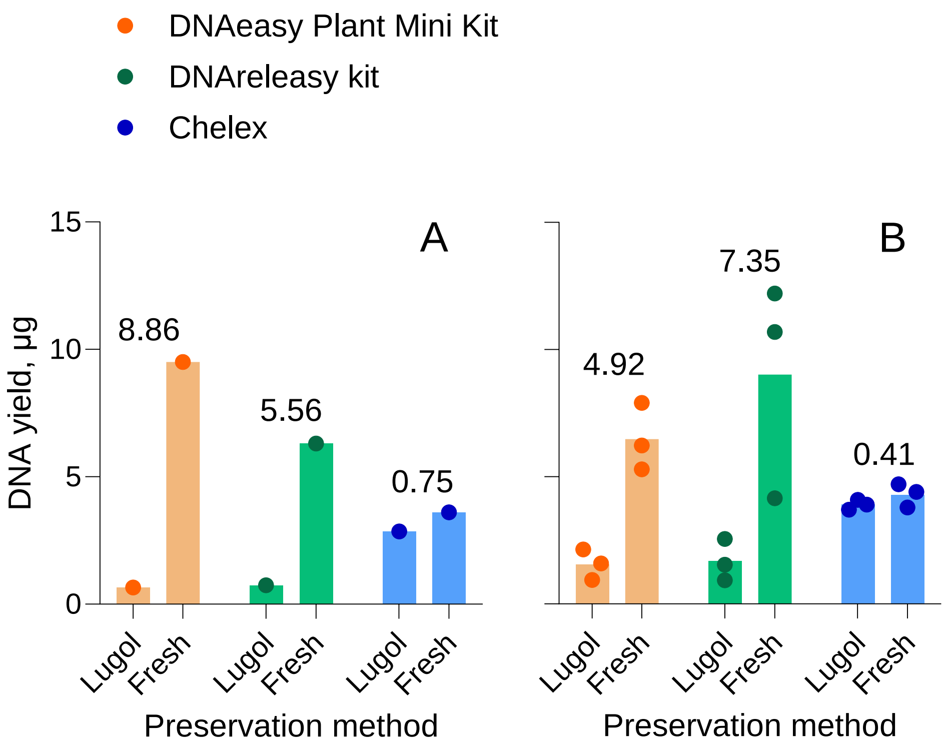

Supplementary Figure 2. Comparison between three different DNA extraction protocols applied to the Lugol-preserved and fresh samples of (A) GCTV-B4 culture (15 mL), and (B) field-collected plankton (25 mL). Bars indicate average values and dots are sample-specific values. The numbers on top are the Euclidean distances between the Lugol-preserved and fresh samples for the same extraction protocol.

II. Cell recovery from the filter

Methods. We used 5.0 µm Millipore TMTP Isopore polycarbonate membrane filter (Ø 25 mm) (Millipore, MA, USA) to collect algal cells (25 mL) from the very dense culture, mimicking algal bloom. To minimize the amount of residual DNA left on the membrane filter after the cell harvesting, i.e. DNA losses in the harvest, we refined the extraction method by cutting the filter into smaller pieces and extracting DNA with these filter pieces still in the tube (*Cut-filter* method). This procedure was then compared with the original extraction method (Penna et al., 2006; Penna and Galluzzi, 2013), where cells detached from the whole filter were then subjected to DNA extraction with the filter removed (*Removed-filter* method).

Cell harvesting was conducted using 1 mL nuclease-free water (H2O) added to the tube with the filter and vortexed to detach the cells. After spinning for 5 min at 10,000 rpm, 800 µL (cell-free) supernatant were removed (leaving 200 µL for extraction). The filter was cut in 8 pieces, transferred to another tube, where 100 µL H2O were added and the DNA extraction proceeded in parallel.

Results. The outcome of the tests is presented in **Supplementary Figure 3A**. The DNA amount was lower for the *Removed-filter* method, i.e., when only detached cells contributed to the DNA yield. Moreover, the variability between the replicates for the latter method was higher compared to the *Cut-filter* method (Coefficient of variation, CV%: 24.6% vs 5.7%). Even though the difference in the mean value was not statistically significant (due to the limited number of replicates; n = 3), the biological difference is substantial and justifies using the *Cut-filter* method to ensure a more complete extraction.

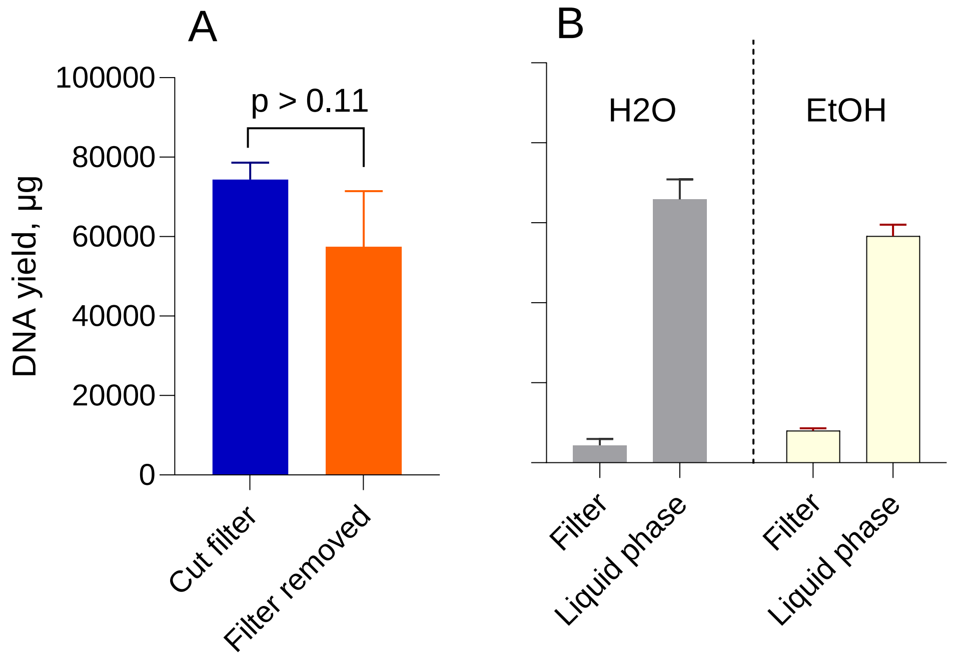

Supplementary Figure 3. Comparison between the two filter handling procedures (A) and (B) cell washing media (water vs ethanol) using the culture of GCTV-B4 (25 mL). Data are shown as mean and SD (n = 3 in all cases). See **Supplementary Table 3** for a statistical evaluation of the effects.

Conclusion. To minimize cell losses due to the partial detachment of the plankton cells from the filter, we recommend cutting the filter and extracting DNA with the filter pieces still in the tube during the extraction process.

III. Cell harvesting media

Methods. Nuclease-free water and ethanol (EtOH) are conventionally used for recovering cells collected on filters for DNA extraction. We compared these two media in terms of their capacity to affect the DNA yield in the liquid phase and the residual DNA amount associated with the filter. The treatment with water proceeded as described in the previous section, whereas, in the EtOH treatment, we added 1 mL of EtOH instead of the water when collecting the cells from the filter. Then, the EtOH was evaporated and 200 µL H2O were added to proceed with the DNA extraction in the same way as in the H2O treatment described above.

Results. The outcome of the tests is presented in **Supplementary Figure 3B** and **Supplementary Table 3**. The effects of media (H2O vs EtOH) and compartment (liquid phase vs filter) were evaluated by a 2-way ANOVA. The DNA yield was significantly affected by both variables; moreover, the interaction term was significant, meaning that in the H2O treatment, significantly less DNA was associated with the filter compared to the EtOH treatment. This finding, together with the significantly higher DNA yield in the H2O treatment, suggests water as a superior medium for cell harvesting.

Conclusion. To maximize cell harvest from the filter, we recommend using nuclease water and not ethanol for recovering filter-collected plankton cells.

Supplementary Table 2. DNA extraction protocols and the kits/products used.

Method/kit	A260/A280	General comments	Manufacturer and link to the kit/product
Protocol 1. DNeasy Plant Mini Kit	1.65-1.78	well-established method known for its high-purity DNA yield but involves multiple steps, which can increase variability.	Qiagen; https://www.qiagen.com/us/resources/resourcedetail?id=6b9bcd96-d7d4-48a1-9838-58dbfb0e57d0&lang=en
Protocol 2. DNAreleasy	1.45-1.67	simpler method designed for quick DNA release, though it may yield lower DNA quality compared to other methods.	Anachem (now available at Nippongenetics); https://www.nippongenetics.eu/en/products/pcr-reagents-and-enzymes/endpoint-pcr/direct-pcr/dnareleasy-advance-1-5-ml-50-rxns/
Protocol 3. Chelex	1.46-1.73	a streamlined process with fewer steps, leading to reduced variability and a quicker procedure, while still maintaining high DNA efficiency.	Bio-Rad; https://www.bio-rad.com/en-se/product/chelex-100-molecular-biology-grade-resin?ID=bd6fd35e-c8ff-4515-9499-8af8ccbb3ca5

Supplementary Table 3. Two-way ANOVA output for the effects of media (water vs ethanol) used for cell recovery from the filter samples. The DNA was extracted from two compartments (filter and liquid phase), and the yields were compared between the compartments and the media. Kolmogorov-Smirnov test was used to evaluate the normality of the distribution for the residuals (distance: 0.2300; $p < 0.079$). See **Supplementary Figure 3B** for visualization.

ANOVA table	SS	DF	MS	F (DFn, DFd)	p value
Interaction	1.012	1	1.012	F (1, 8) = 13.96	p=0.0057
Compartment	34.56	1	34.56	F (1, 8) = 476.4	p<0.0001
Media	0.4004	1	0.4004	F (1, 8) = 5.519	p=0.0467
Residual	0.5804	8	0.07255		

References

Bowers, H. A., Tengs, T., Glasgow, H. B., Burkholder, J. M., Rublee, P. A. and Oldach, D. W. (2000). Development of real-time PCR assays for rapid detection of Pfiesteria piscicida and related dinoflagellates. Appl. Environ. Microbiol. 66, 4641–4648. doi:10.1128/aem.66.11.4641-4648.2000

Dyhrman, S. T., Erdner, D., Du, J. L., Galac, M. and Anderson, D. M. (2006). Molecular quantification of toxic Alexandrium fundyense in the Gulf of Maine using real-time PCR. Harmful Algae 5, 242–250. doi:10.1016/j.hal.2005.07.005

Eckford-Soper, L.K., Daugbjerg, N., 2015. Examination of six commonly used laboratory fixatives in HAB monitoring programs for their use in quantitative PCR based on Taqman probe technology. Harmful Algae 42, 52–59. https://doi.org/10.1016/j.hal.2014.12.007

Engesmo, A., Strand, D., Gran-Stadniczeñko, S., Edvardsen, B., Medlin, L. K. and Eikrem, W. (2018). Development of a qPCR assay to detect and quantify ichthyotoxic flagellates along the Norwegian coast, and the first Norwegian record of Fibrocapsa japonica (Raphidophyceae). Harmful Algae 75, 105–117. doi:10.1016/j.hal.2018.04.007.

Hernández-Becerril, D. U., Lau, W. L. S., Hii, K. S., Leaw, C. P., Varona-Cordero, F. and Lim, P. T. (2018). Abundance and distribution of the potentially toxic thecate dinoflagellate Alexandrium tamiyavanichii (Dinophyceae) in the central Mexican Pacific, using the quantitative PCR method. Front. Mar. Sci. 5. doi:10.3389/fmars.2018.00366.

Kon, N. F., Teng, S. T., Hii, K. S., Yek, L. H., Mujahid, A., Lim, H. C., Lim, P. T. and Leaw, C. P. (2015). Spatial distribution of toxic Alexandrium tamiyavanichii (Dinophyceae) in the southeastern South China Sea-Sulu Sea: A molecular-based assessment using real-time quantitative PCR (qPCR) assay. Harmful Algae 50, 8–20. doi:10.1016/j.hal.2015.10.002.

Motwani, N.H., Gorokhova, E., 2013. Mesozooplankton Grazing on Picocyanobacteria in the Baltic Sea as Inferred from Molecular Diet Analysis. PLoS ONE 8, e79230. https://doi.org/10.1371/journal.pone.0079230

Penna, A., Bertozzini, E., Battocchi, C., Galluzzi, L., Giacobbe, M.G., Vila, M., Garces, E., Luglie, A., Magnani, M., 2006. Monitoring of HAB species in the Mediterranean Sea through molecular methods. J. Plankton Res. 29, 19–38. https://doi.org/10.1093/plankt/fbl053

Penna, A., Galluzzi, L., 2013. The quantitative real-time PCR applications in the monitoring of marine harmful algal bloom (HAB) species. Environ. Sci. Pollut. Res. Int. 20, 6851–6862. https://doi.org/10.1007/s11356-012-1377-z

Ruvindy, R., Bolch, C. J., MacKenzie, L., Smith, K. F. and Murray, S. A. (2018). qPCR assays for the detection and quantification of multiple paralytic shellfish toxin-producing species of Alexandrium. Front. Microbiol. 9. doi:10.3389/fmicb.2018.03153

Smith, K. F., de Salas, M., Adamson, J. and Rhodes, L. L. (2014). Rapid and accurate identification by real-time PCR of biotoxin-producing dinoflagellates from the family Gymnodiniaceae. Mar. Drugs 12, 1361–1376. doi:10.3390/md12031361

Stern, R. F., Andersen, R. A., Jameson, I., et al. (2012). Evaluating the ribosomal internal transcribed spacer (ITS) as a candidate dinoflagellate barcode marker. PLoS ONE 7, e42780. doi:10.1371/journal.pone.0042780

Toebe, K., Joshi, A. R., Messtorff, P., Tillmann, U., Cembella, A. and John, U. (2013). Molecular discrimination of taxa within the dinoflagellate genus Azadinium, the source of azaspiracid toxins. J. Plankton Res. 35, 225–230. doi:10.1093/plankt/fbs077
